# LSTM Short-Term Wind Power Prediction Method Based on Data Preprocessing and Variational Modal Decomposition for Soft Sensors

**DOI:** 10.3390/s24082521

**Published:** 2024-04-15

**Authors:** Peng Lei, Fanglan Ma, Changsheng Zhu, Tianyu Li

**Affiliations:** 1 Network & Information Center, Lanzhou University of Technology, Lanzhou 730050, China; 2 School of Computer and Communication, Lanzhou University of Technology, Lanzhou 730050, China; 3 Institute of Sensing Technology, Gansu Academy of Sciences, Lanzhou 730000, China

**Keywords:** short-term wind power prediction, LSTM, VMD, data preprocessing, isolation forest, soft sensor

## Abstract

Soft sensors have been extensively utilized to approximate real-time power prediction in wind power generation, which is challenging to measure instantaneously. The short-term forecast of wind power aims at providing a reference for the dispatch of the intraday power grid. This study proposes a soft sensor model based on the Long Short-Term Memory (LSTM) network by combining data preprocessing with Variational Modal Decomposition (VMD) to improve wind power prediction accuracy. It does so by adopting the isolation forest algorithm for anomaly detection of the original wind power series and processing the missing data by multiple imputation. Based on the process data samples, VMD technology is used to achieve power data decomposition and noise reduction. The LSTM network is introduced to predict each modal component separately, and further sum reconstructs the prediction results of each component to complete the wind power prediction. From the experimental results, it can be seen that the LSTM network which uses an Adam optimizing algorithm has better convergence accuracy. The VMD method exhibited superior decomposition outcomes due to its inherent Wiener filter capabilities, which effectively mitigate noise and forestall modal aliasing. The Mean Absolute Percentage Error (MAPE) was reduced by 9.3508%, which indicates that the LSTM network combined with the VMD method has better prediction accuracy.

## 1. Introduction

The power output of a wind power generation system has great randomness, volatility and uncertainty, so its large-scale integration into the power grid brings great challenges to the safe and stable operation of the power system [1,2]. Soft sensor technology establishes a mathematical model between the auxiliary and target variables to predict the target variables, which has the characteristics of low cost and high accuracy [3]. However, strongly nonlinear, dynamic time-varying and multi-rate data characteristics are caused by poor soft sensor performance. Short-term forecasting of wind power using deep learning can provide a reference for the dispatch of the intraday power grid and is one of the key technologies in solving the above-mentioned problems [4].

At present, the related research on short-term wind power forecasting mainly focuses on establishing more accurate forecasting models, data preprocessing and signal decomposition. Many existing prediction models treat input samples as different classes and classify them, which may lead to long-term dependencies between samples that cannot be fully utilized [5]. In addition, human factors, or the failure of wind turbine equipment, may lead to abnormal or missing parts of the power data, which affects the accuracy of wind power prediction. Therefore, in order to ensure the reliability of the data, detecting abnormal values in power data is essential. The use of an unsupervised isolated forest algorithm based on an ensemble learning strategy to detect abnormal data can effectively improve data processing efficiency and improve the accuracy of abnormality detection [6]. VMD is a new type of signal decomposition method which can decompose the signal into several modal components according to the potential characteristics of wind power, and VMD has less parameter settings, good robustness, high computational efficiency and a rich theoretical basis [7,8,9]. Using the VMD method can make the input data of the prediction model reflect the characteristics of the wind power signal more clearly. A new hybrid wind speed forecasting model using Variational Modal Decomposition (VMD), the partial autocorrelation function (PACF), and a weighted regularized extreme learning machine (WRELM) is proposed to improve the accuracy of wind speed forecasting [10].

In recent years, deep learning has dramatically improved the learning ability of neural networks via residual connection and shared weights, which are widely used in dynamic soft sensor modeling. A Recurrent Neural Network (RNN) [11] has recursive links in its network structure, and the relationship between samples can be considered in the learning process, so it is especially suitable for processing time series signals. But if there are long-term dependencies between samples, RNNs will suffer from vanishing gradients and exploding gradients. A Long Short-Term Memory (LSTM) network is an improved method for addressing this problem. In recent years, LSTM has been used more and more in wind power [12,13,14,15]. Yu et al. [16] combined wavelet transforms to establish a new hybrid model based on three recurrent neural networks. The results demonstrate that the three new hybrid models produce more accurate prediction results. Nevertheless, it is poor adaptability that the decomposition effect of the wavelet transform depends on the choice of threshold and the basis function. Curreri et al. [17] compared the recurrent neural networks and long short-term memory architectures in regard to their transferability. The obtained results demonstrate the suitability of the proposed transfer learning methods in the design of nonlinear dynamical models for industrial systems. Zhang et al. [18] proposed a strategy of building a soft sensor model based on local semi-supervised ensemble learning of least squares support vector regression, which is used to deal with nonlinear, dynamic time-varying and multi-rate data regression problems in wind power generation processes. Han et al. [19] used the VMD technique to decompose the original wind power signal and used the decomposed components as the input of the improved LSTM prediction model to predict the wind power. However, data preprocessing operations, such as outlier detection of the collected raw data, are ignored. Aiming at the above problems, this paper combines the forecasting model and wind power data processing to improve forecasting accuracy and proposes a LSTM short-term wind power forecasting model based on data preprocessing and VMD [20] for the soft sensor. We use the isolation forest and multiple imputation methods to deal with outliers and missing values of wind power data [21].

An LSTM network consists of an input layer, an output layer, and several recursive hidden layers between them. The recursive hidden layers are composed of several memory modules. Each module contains one or more self-connected memory cells and three gates that control the flow of information: memory gates, forgetting gates and output gates [19].

To minimize the sum of frequency bandwidths to complete noise reduction, VMD technology is adopted to decompose historical power data into several modal components. The LSTM method is imported to establish a prediction model for each modal component. The Dropout parameter regularization method is used to establish the model to prevent over-fitting. The Adam algorithm is adopted to optimize the effective training of the network parameters of the LSTM model. Ultimately, sum and reconstruct are used to predict the results of the components. Compared to the results of BP (Back Propagation), SVM (Support Vector Machine), LSTM and Complete Ensemble Empirical Mode Decomposition-LSTM (CEEMDAN-LSTM) models, the experimental results indicate that the VMD-LSTM model has higher prediction accuracy.

## 2. Method

### 2.1. VMD

VMD [22] decomposes the signal $f\left(t\right)$ into discrete modal components $u_{k}\left(k=1,\;2,\;3,\cdots ,\;K\right)$; for each modal component $u_{k}$, the Hilbert transform is used to calculate the relevant analytic signal and obtain the unilateral spectrum, where $\delta \left(t\right)$ is the pulse function.

(1) $$U(t)=\left(\delta \left(t\right)+\frac{j}{\pi t}\right)\times u_{k}\left(t\right)$$

The analytical signal obtained from Equation (1) above is mixed with the estimated center frequency $e^{-j\omega_{k}t}$, and the spectrum corresponding to each mode is transformed to the corresponding baseband.

(2) $$\hat{U(t)}=U(t)\times e^{-j\omega_{k}t}$$

Calculate the square norm of the gradient of the demodulated signal, obtain the bandwidth of each mode, and then construct the constrained variational problem, where $u_{k}=\left\{u_{1},\cdots ,u_{k}\right\}$, $\omega_{k}=\left\{\omega_{1},\cdots ,\omega_{k}\right\}$.

(3) $$L2=\left\{\begin{array}{l} \underset{(u_{k}),\left(\omega_{k}\right)}{min}\left\{{\left\|\partial_{t}\hat{U(t)}\right\|}^{2}\right\}\\ s.t.\sum_{k=1}^{K}u_{k}=f \end{array}\right.$$

The augmented Lagrangian function is introduced to find the optimal solution for the constrained variational problem, where α is the quadratic penalty factor, and λ(t) is the Lagrangian multiplication operator.

(4) $$L\left(\left\{u_{k}\right\},\left\{\omega_{k}\right\},\lambda \right)=\alpha \sum_{k=1}^{K}{\left\|\partial_{t}\hat{U(t)}\right\|}_{2}^{2}+{\left\|f(t)-\sum_{k=1}^{K}u_{k}(t)\right\|}_{2}^{2}+\left\langle \lambda (t),f(t)-\sum_{k=1}^{K}u_{k}(t)\right\rangle$$

The alternating direction multiplier method (ADMM) is used to update ${\hat{u}}_{k}^{n+1}$ and $\omega_{k}^{n+1}$ to find the optimal solution for Equation (3).

(5) $${\hat{u}}_{k}^{n+1}\left(\omega \right)=\frac{\hat{f}\left(\omega \right)-\sum_{i\neq k}{\hat{u}}_{i}\left(\omega \right)+\frac{\hat{\lambda }\left(\omega \right)}{2}}{1+2\alpha {\left(\omega -\omega_{k}\right)}^{2}}\;\;\;$$

(6) $$\omega_{k}^{n+1}=\frac{\int_{0}^{\infty }\omega {\left|{\hat{u}}_{k}\left(\omega \right)\right|}^{2}d\omega }{\int_{0}^{\infty }{\left|{\hat{u}}_{k}\left(\omega \right)\right|}^{2}d\omega }\;\;\;$$

where ${\hat{u}}_{k}^{n+1}\left(\omega \right)$, $\hat{f}\left(\omega \right)$, ${\hat{u}}_{i}\left(\omega \right)$ and $\hat{\lambda }\left(\omega \right)$ are Fourier transforms of $u_{k}^{n+1}\left(t\right)$, $f\left(t\right)$ and $\lambda \left(t\right)$, respectively.

### 2.2. LSTM

**Figure 1:**
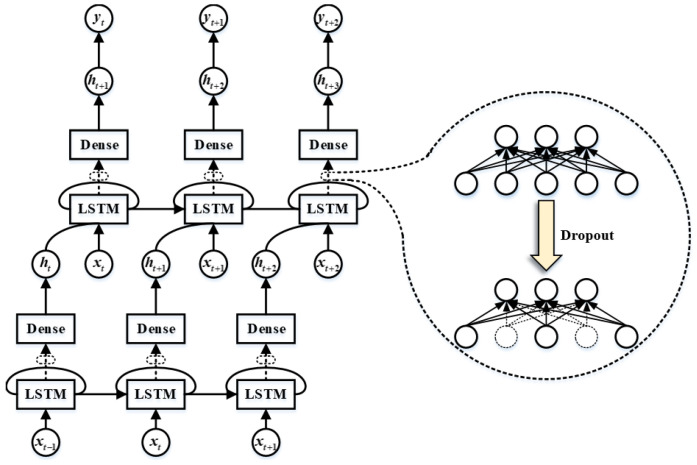
The structure of LSTM.

The LSTM memory unit is used to build the LSTM [23] network prediction model, and the Dropout regularization method [24] is used between the hidden layer and the Dense Layer to prevent over-fitting and improve the model generalization ability. The LSTM model network structure is shown in Figure 1. Among them, LSTM represents the LSTM memory unit, and Dense represents the full connection layer. When Dropout is applied to the LSTM layer, the input of the Dense layer is the output of the LSTM layer. This process is represented by dotted arrows and dotted circles to illustrate the principle of the Dropout method. The input in the t−1 time model is represented as the variable $x_{t-1}$. After the Dropout method, the LSTM layer outputs the results to the Dense layer, and the corresponding representation is $h_{t}$. If $x_{t}$ and $h_{t}$ are the inputs at the next time t+1, the output can be represented as $h_{t+1}$ after the Dropout method, where α is the quadratic penalty factor, and λ(t) is the Lagrangian multiplication operator.

In addition, the Adam algorithm is used to optimize LSTM network parameters and train the network model. The updated rules of the Adam algorithm optimization parameters are as follows:

Calculate the gradient when t, and initialize $t_{0}=0$ where $J\left(\theta \right)$ is the random objective function of θ.

(7) $$g_{t}=\nabla_{\theta }J\left(\theta_{t-1}\right)\;$$

Calculate the biased first moment estimate $m_{t}$ of the gradient and initialize $m_{0}=0$ where parameter $\beta_{1}=0.9$.

(8) $$m_{t}=\beta_{1}m_{t-1}+\left(1-\beta_{1}\right)g_{t}\;\;$$

Calculate the biased first moment estimate $v_{t}$ of the gradient and initialize $v_{0}=0$ where parameter $\beta_{2}=0.999$.

(9) $$\;v_{t}=\beta_{2}v_{t-1}+\left(1-\beta_{2}\right)g_{t}^{2}\;\;$$

Correct the deviation of the first order moment estimation and express the result as $\hat{m_{t}}$.

(10) $$\hat{m_{t}}=\frac{m_{t}}{1-\beta_{1}^{t}}\;\;\;$$

Correct the deviation of the first order moment estimation and express the result as $\hat{v_{t}}$.

(11) $$\hat{v_{t}}=\frac{v_{t}}{1-\beta_{2}^{t}}\;$$

Calculate parameter θ at time t.

(12) $$\theta_{t}=\theta_{t-1}-\frac{\alpha \hat{m_{t}}}{\sqrt{\hat{v_{t}}}+\varepsilon }\;$$

## 3. Results and Analysis

In this section, we first describe the real-world dataset used in the experiments. Then, we discuss the experiments conducted on the dataset.

### 3.1. Experimental Data and Evaluation Indicators

To evaluate our method, we performed anomaly detection and missing value interpolation operations on 17,280 sets of data with a sampling interval of 1 min from 20 May to 31 May 2015 at a wind farm in Shanxi Province, China. We used the 12 day data as the experiment’s dataset. Each datum in the dataset is a two-dimensional datum which includes the wind speed and the wind farm. Then, taking every 15 sets of data as a cycle, that is, a cycle of 15 min, the average value of the data within 15 min is recorded, and the 1152 sets of data recorded are used as wind power data research samples and used for experimental analysis. Following chronological order, we take the first 1056 sets of data as the training sets and forecast the wind power for 15 min in the future. After each step of forecasting is achieved, the predicted value is used instead of the actual value to perform the iterative calculation of the next forecast. The forecast sequence is 1058–1151, a total of 94 wind power data for each data point. The experimental data is processed by the min-max normalization (MMN) method for dimensionless data processing, and the data values are mapped to the [0, 1] interval. After the prediction is completed, the predicted value is converted to the original interval by inverse normalization.

In the experiment, we chose Root Mean Squared Error (*RMSE*), Mean Absolute Error (*MAE*), Mean Absolute Percentage Error (*MAPE*), and Relative Error (*RE*) as an evaluation index for the prediction results. $y_{i}$ represents the actual value of wind power, $\hat{y_{i}}$ represents the predicted value of wind power, N represents the wind power sequence data amount, and $\delta_{i}$ represents the relative error.

(13) $$RMSE=\sqrt{\frac{1}{N}{\sum_{i=1}^{N}\left(y_{i}-\hat{y_{i}}\right)}^{2}}\;\;\;\;\;$$

(14) $$MAE=\frac{1}{N}\sum_{i=1}^{N}\left|y_{i}-\hat{y_{i}}\right|\;\;$$

(15) $$MAPE=\left(\frac{1}{N}\sum_{i=1}^{N}\left|\frac{y_{i}-\hat{y_{i}}}{y_{i}}\right|\right)$$

(16) $$\delta_{i}=\frac{\left|y_{i}-\hat{y_{i}}\right|}{y_{i}}\times 100\%\;\;$$

### 3.2. Data Preprocessing and Result Analysis

In this work, the isolated forest algorithm is used to detect abnormal wind power data. After detection by the algorithm, the abnormal data are marked as one, and the non-abnormal data are marked as zero. All abnormal data are set to zero according to the index number of identifier one, and the complete data set is further obtained by the method of multiple imputation.

**Figure 2:**
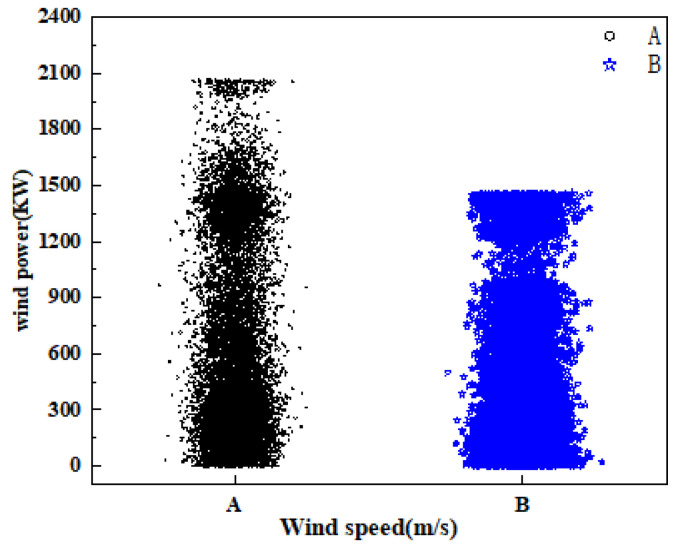
Comparison of histograms during data processing.

The columnar scatter diagram before and after processing the raw wind power data is shown in Figure 2. The horizontal axis represents wind speed (m/s); part A in the figure represents the collected 17,280 groups of original wind power data, and part B represents the wind power data after data preprocessing. The vertical axis is wind power (KW). It can be seen that the data distribution of the unprocessed part A is highly random and chaotic, and the data points located in the power value range above 1500 KW deviate from most of the data points. After processing, the data distribution of part B is concentrated and the data points at both ends of the cylinder center are compact and orderly, which effectively enhances the reliability and integrity of the data. The processed data are decomposed into K modal components using VMD signal decomposition technology, and the value of K is determined by observing the center frequency distribution corresponding to each component under different modal numbers. In Table 1, when the number of modes is five, the corresponding center frequencies of *IMF2*, *IMF3* and *IMF4* are 78.47 Hz, 123.54 Hz and 396.97 Hz, respectively, and the center frequencies are close to each other, which indicates that modal aliasing may occur, resulting in over-resolution of the wind power signal.

**Figure 3:**
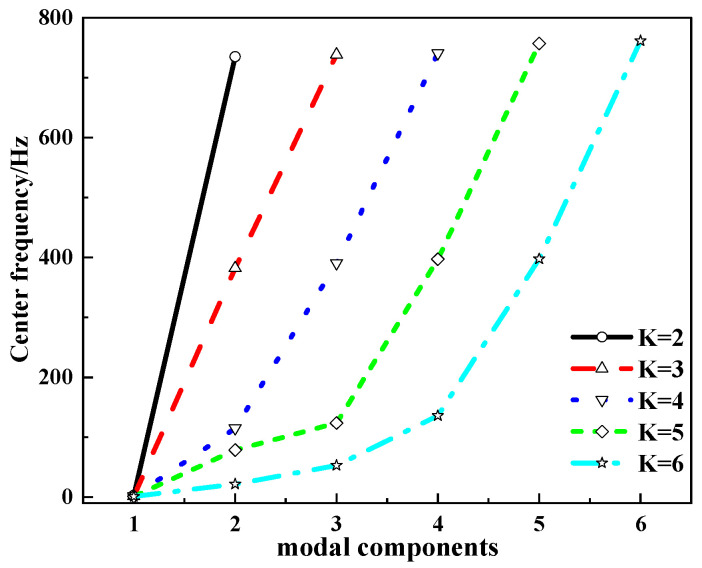
Change curve of center frequency of different modal components.

To determine the value of *K*, we analyzed the change curve of the center frequency of each component, which is shown in Figure 3. It can be seen from Figure 3 that when *K* = 2, *K* = 3 and *K* = 4, the curves have no obvious curvature, and the slope changes little. When *K* = 5, the curve shows an obvious downward bending phenomenon, and the change of curve bending indicates that when *K* is the critical value of the modal decomposition number, the critical value is considered to be an appropriate modal decomposition number.

In order to further determine the value of K, the Pearson correlation coefficient of adjacent modal components is calculated, and Table 2 analyzes the correlation between adjacent modal components. Among them, $C_{12}$ represents the Pearson correlation coefficient between *IMF1* and *IMF2*, $C_{23}$ represents the correlation coefficient between *IMF2* and *IMF3*, and the same is true for $C_{34}$, $C_{45}$ and $C_{56}$. When K<5, the values of $C_{12}$, $C_{23}$ and $C_{34}$ are all less than 0.1, indicating that the low- and high-frequency components obtained by signal decomposition have obvious ${\begin{array}{c} C \end{array}}_{12}$ characteristics. When K=5, $C_{12}$ = 0.3614, $C_{23}$ = 0.2906 and the correlation coefficient is relatively large, which indicates that the low-frequency modal components obtained by decomposition are highly correlated, the signal decomposition is not sufficient, and modal aliasing is prone to occur.

In Table 2, the Pearson correlation coefficient of adjacent modes based on the above analysis determines *K* = 4, and sets parameters *α* = 2000 and *ε* = 0.000001, where *α* is the quadratic penalty factor, and *ε* is the convergence accuracy.

**Figure 4:**
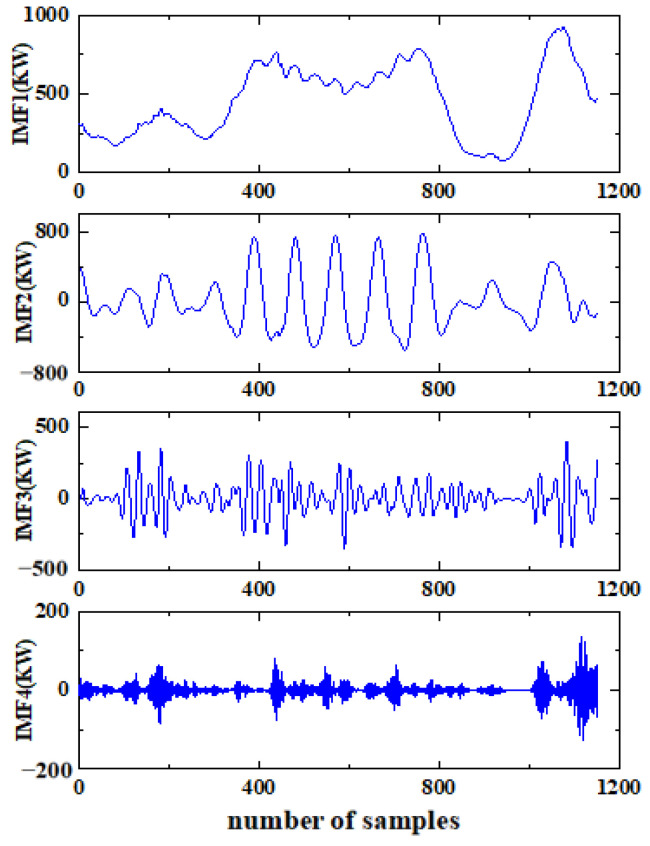
Results of VMD decomposition.

The results of decomposing the wind power signal sequence by VMD are shown in Figure 4. Among them, *IMF1* to *IMF4* are the decomposed wind power sequence components from low frequency to high frequency.

**Figure 5:**
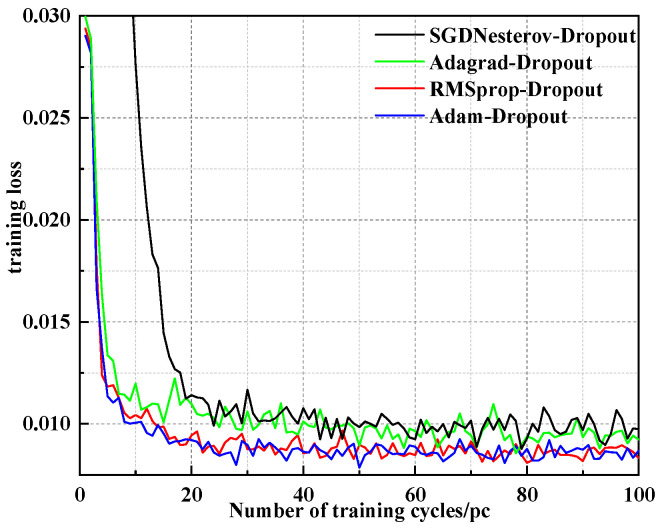
Training algorithm contrast curve.

The RMSProp (Root Mean Square prop) algorithm, Adagrad (Adaptive gradient) algorithm, SGDNesterov (Stochastic Gradient Descent Nesterov) algorithm and Adam (Adaptive Moment Estimation) algorithm are combined with the Dropout regularization method to train the LSTM network model. The input data is the preprocessed wind power sequence without VMD decomposition; the parameter p=0.3 in the Dropout method is set, and the Mean Square Error (*MSE*) function is constructed as the loss function. The different curves in Figure 5 represent the change of the loss function in each training model, respectively. It can be seen from the figure that Adam is significantly faster than the SGDNesterov algorithm in terms of convergence speed. Adam, RMSProp and Adagrad have basically the same convergence speed, but in the 50th training cycle, Adam has the lowest training loss value, which is only 0.0078, indicating that the algorithm can achieve better convergence accuracy.

The wind power sequence is decomposed by VMD, and then the LSTM network prediction model is established, respectively, to predict each IMF component and obtain the prediction result, as shown in Figure 5. In the LSTM network model, the input layer is one layer, the hidden layer is one layer, the hidden layer contains 16 LSTM neurons, the fully connected layer is one layer, the number of output layers is one, the loss function is *MSE*, and the optimization algorithm is Adam. Add the Dropout regularization method and set probability between the hidden layer and fully connected layer. The prediction results of each component are summed and reconstructed, and finally the prediction results of the VMD-LSTM [25,26,27,28] model are obtained. The LSTM model is used to predict the wind power sequence without VMD, and the prediction result of the LSTM model is obtained.

**Figure 6:**
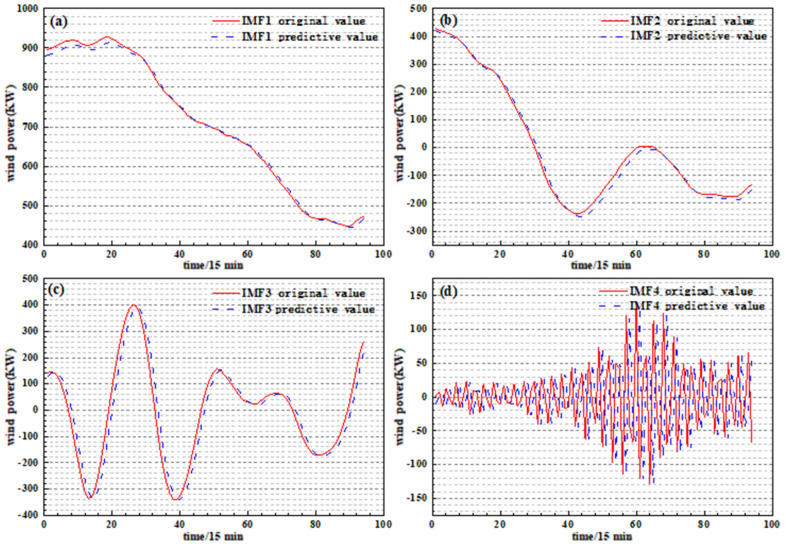
Prediction results of each IMF component: (**a**) *IMF1*; (**b**) *IMF2*; (**c**) *IMF3*; (**d**) *IMF4*.

**Figure 7:**
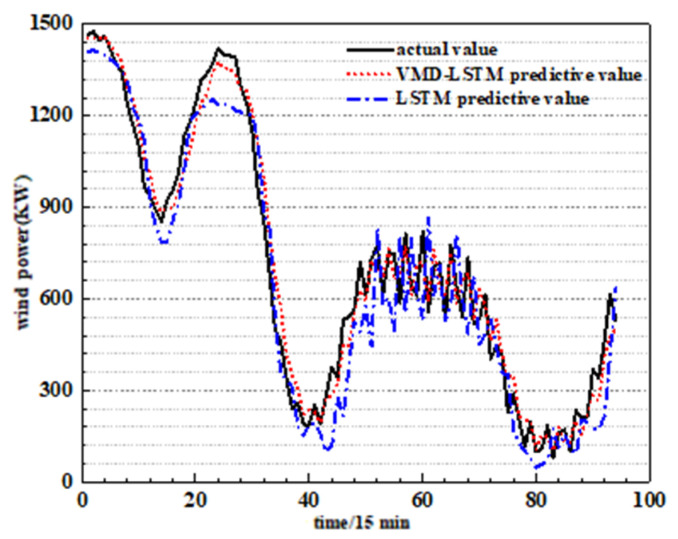
Prediction results of VMD-LSTM.

The comparison curve between the predicted value of the VMD-LSTM model and LSTM model and the actual wind power value is shown in Figure 6. It can be seen from Figure 7 that for the LSTM prediction model, the prediction results roughly follow the change trend of the actual wind power sequence, but when the actual power value suddenly changes, the prediction effect is poor. However, the error between the predicted value of the VMD-LSTM model and the actual value is small, and the trend change is completely consistent. Therefore, using VMD to decompose the original power signal can effectively extract data features and deal with fluctuations in wind power information.

In this study, we use CEEMDAN-LSTM, BPNN and SVM to compare and analyze the performance of the VMD-LSTM model. In this experiment, it is assumed that there are 500 groups of white noise signals with a standard deviation of 0.2 in the CEEMDAN signal decomposition, and the maximum number of iterations is 5000. In the BPNN, the number of neurons in the input layer is one, the number of neurons in the hidden layer is 15, the number of neurons in the output layer is one, the learning rate is 0.01, and the maximum number of trainings is 100. Using grid method cross-validation in SVM, the parameters are 12.32 and 22.05.

**Figure 8:**
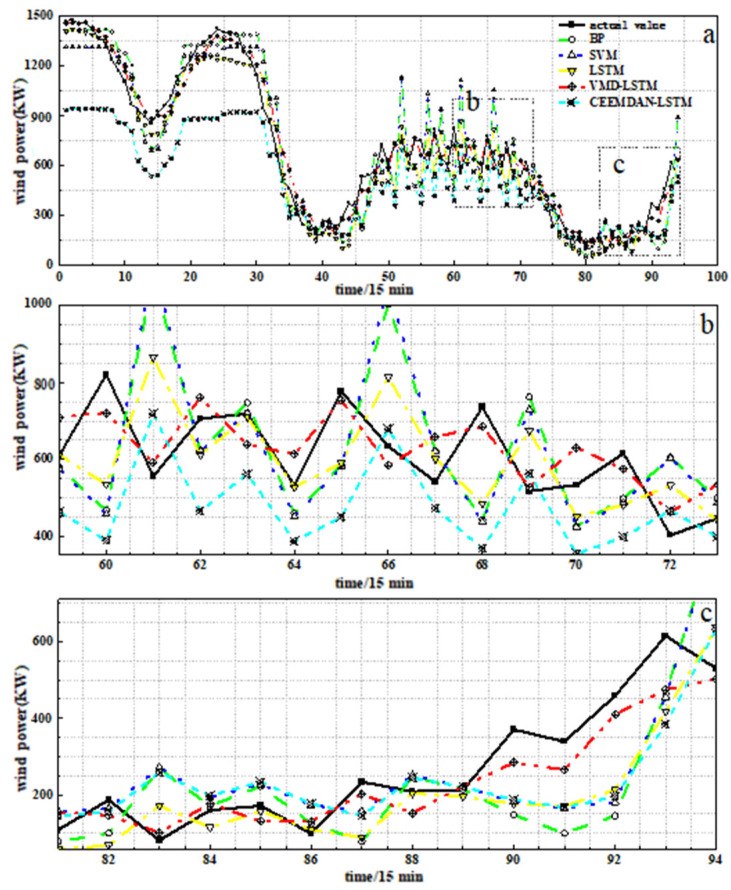
Wind power prediction curve: (**a**) comparison of prediction results among different models; (**b**) partial amplification of each mode; (**c**) partial amplification of each mode.

Figure 8a is a dotted line graph of the prediction results of each model; the actual value represents the actual wind power, and Figure 8b,c are partial enlarged comparisons of each model. It can be seen from Figure 8 that the wind power prediction curves of BPNN, SVM and LSTM are consistent with the trend of the actual wind power series, but the prediction results of the LSTM model are closer to the actual value. This proves that the LSTM model can effectively capture the long-term dependencies between data samples, and the LSTM model is suitable for processing time series signals. Meanwhile, we can see that the CEEMDAN-LSTM model has a poor prediction effect and large prediction error. The VMD-LSTM model can not only accurately describe the dynamic changes of the original wind power sequence, but also the predicted value is closest to the actual wind power value. This shows that compared with CEEMDAN, the VMD method with a Wiener filter can effectively remove signal noise, can distinguish between effective signal information and noise signal significantly and has strong robustness. By decomposing the original wind power series through VMD, more accurate prediction results can be obtained, thereby improving the prediction performance of the model.

**Figure 9:**
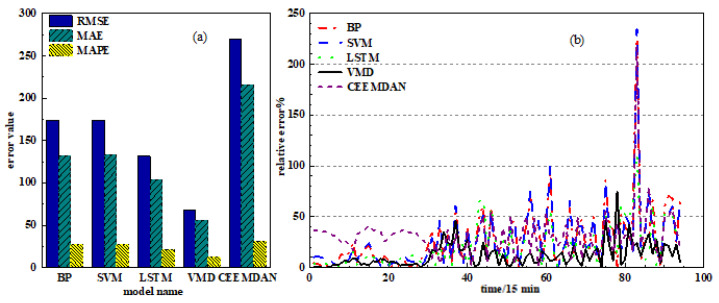
Comparison of prediction errors between models: (**a**) model error histogram; (**b**) model error curve.

In order to further reflect the prediction error at each moment, as shown in Figure 9, we calculate the relative error of each model and draw its relative error curve to visualize the error evaluation index of each model. Figure 9a is a columnar schematic diagram of the three error evaluation indicators of the model: *RMSE*, *MAE* and *MAPE*. It can be seen that the *RMSE*, *MAE* and *MAPE* values of the CEEMDAN-LSTM model are the largest, and the *RMSE*, *MAE* and *MAPE* values of the VMD-LSTM model are significantly lower than the error values of the other models. From the relative error curves of each model in Figure 9b, it can be seen that except for the relative error of the 78th prediction point, which is 75%, the relative errors of the remaining prediction points of the VMD-LSTM model are all below 50%. Especially, the prediction accuracy at the abrupt point of wind speed is significantly improved compared with other models, which indicates that the VMD-LSTM method can improve the accuracy of wind power prediction.

Table 3 lists the specific values of the error evaluation indicators in each prediction model. It can be seen from Table 3 that the *RMSE*, *MAE* and *MAPE* of the VMD-LSTM model are 67.6993, 55.7662 and 12.0676, respectively, which has the smallest prediction error compared with other models. Compared with the single prediction models BPNN and SVM, the *RMSE* of VMD-LSTM is reduced by 106.1781 and 106.3299, respectively. After using the decomposition algorithm, the *MAPE* value of CEEMDAN-LSTM is increased by 10.04685% compared with LSTM, and the *MAPE* value of VMD-LSTM is decreased by 9.3508% compared with LSTM. Meanwhile, the *RMSE* and *MAE* values of VMD-LSTM are reduced by 63.9672 and 48.1798, respectively, compared with LSTM. It can be verified that the VMD-LSTM model has good prediction performance and can effectively improve the prediction accuracy of wind power, which is suitable for short-term prediction of actual wind power.

## 4. Conclusions

In this study, we proposed a LSTM short-term wind power prediction model based on isolated forest outlier detection and VMD for the soft sensor. In the model, the isolated forest algorithm is used to detect the outliers of the wind power series, and the missing values of the data are processed by the multiple imputation method. Denoising decomposition of VMD is performed on the research samples, and the number *K* of modal components is determined according to the center frequency and the correlation coefficient. The LSTM contains a memory unit that can store and update information for a long time, making the network more robust and accurate when processing long time sequences. Additionally, the VMD decomposes the wind power data into K model components, which are used as the inputs of the forecast model. And the LSTM short-term wind power prediction model is established which uses Dropout to prevent over-fitting, and uses the Adam algorithm to optimize the model. The experiment results verify that the model can improve the accuracy of short-term power prediction. In addition, through comparative experiments, we can draw the following conclusions:

1. Using the isolated forest algorithm to detect anomalies in the original wind power sequence and to perform multiple imputation processing on missing data.
2. In terms of data processing, the experimental data is processed using the minimum-maximum normalization (MMN) method for dimensionless data, and the data values are mapped to the [0, 1] interval, which improves the effectiveness of data processing.
3. Compared with the RMSProp algorithm, Adagrad algorithm and SGD Nesterov algorithm, using the Adam algorithm to optimize LSTM network parameters has better convergence accuracy.
4. The VMD method has better decomposition results than the CEEMDAN method because its own Wiener filter can effectively complete the noise reduction and prevent modal aliasing.
5. Compared with traditional BPNN and SVM, LSTM is suitable for short-term wind power prediction and has better prediction accuracy.

## Figures and Tables

**Table 1:** Center frequency corresponding to different *K*.

Modal Number	Center Frequency/Hz					
	*IMF1*	*IMF2*	*IMF3*	*IMF4*	*IMF5*	*IMF6*
2	2.81	735.23				
3	1.43	382.21	738.86			
4	1.22	114.96	390.34	740.88		
5	1.08	78.47	123.54	396.97	757.22	
6	0.97	21.63	52.69	135.72	397.42	761.53

**Table 2:** Pearson correlation coefficient of adjacent modes.

Modal Number	*C*_12_	*C*_23_	*C*_34_	*C*_45_	*C*_56_
2	0.0915				
3	0.0618	0.0900			
4	0.0519	0.0901	0.0963		
5	0.3614	0.2906	0.0257	0.0810	
6	0.3501	0.2860	0.1284	0.0284	0.0601

**Table 3:** Comparison of the prediction indexes of different prediction methods.

	BP	SVM	LSTM	VMD-LSTM	CEEMDAN-LSTM
*RMSE* (KW)	173.8774	174.0292	131.6665	67.6993	270.0046
*MAE* (KW)	131.4138	133.1894	103.9460	55.7662	215.4398
*MAPE* (%)	26.9278	26.8527	21.4184	12.0676	31.4652

## Data Availability

Due to the data sensitive, the data presented in this article are not available.
